# Clinical Applications of Benzosol

**Published:** 1896-11

**Authors:** 


### CLINICAL APPLICATIONS OF BENZOSOL.

DR. J. V. KOFRON, of Cleveland, O., writing in the *American Therapist*, for June, 1896, lauds benzosol very highly in cases of incipient pulmonary tuberculosis, chronic bronchitis, tubercular diarrhea, chronic gastric catarrh, intestinal catarrh with flatulence and typhoid fever.

Very surprising were the results obtained from the use of benzosol in the treatment of cases of tubercular diarrhea, in which the remedies usually administered proved of little value. In doses of four grains every four hours, it seemed to have an almost specific action in checking the exhausting diarrhea and in improving the digestion and general condition of the patient.

Benzosol is a chemically pure benzoate of guaiacol, containing approximately 54 per cent. of the latter agent. It occurs in the form of a slightly pinkish, finely granular powder, which is entirely devoid of taste or odor; it is practically insoluble in any of the ordinary menstrua and is preferably administered in the form of powder, pill or capsule. Benzosol passes unchanged through the stomach and is split up into its component parts (benzoic acid and guaiacol) by the alkaline juices of the intestine.

This fact accounts for two of its principal clinical advantages :

1. The avoidance of the unpleasant eructions which so frequently occur after the administration of pure creosote or guaiacol, and

2. The liberation of the antiseptic guaiacol in the intestine, where its action is especially desired in the treatment of septic conditions of the intestinal tract attended with fermentation.

